# A mixed methods quality improvement study to implement nurse practitioner roles and improve care for residents in long-term care facilities

**DOI:** 10.1186/s12912-019-0395-2

**Published:** 2020-01-29

**Authors:** Kelley Kilpatrick, Éric Tchouaket, Mira Jabbour, Sylvie Hains

**Affiliations:** 1 Susan E. French Chair in Nursing Research and Innovative Practice, Ingram School of Nursing, McGill University, Montréal, Canada; 2 Centre intégré universitaire de santé et de services sociaux de l’Est-de-l’Île-de-Montréal-Hôpital Maisonneuve-Rosemont (CIUSSS-EMTL-HMR), Montréal, Canada; 3 Department of Nursing, Université du Québec en Outaouais, Saint-Jérôme, Canada; 4 CIUSSS EMTL-HMR, Montréal, Canada; 5 Retired, Ministère de la Santé et des services sociaux du Québec, Québec, Canada

**Keywords:** Advanced practice nursing, Analysis of occurrence, Cohort study, Inter-professional team, Long-term care, Mixed methods, Model of care, Nurse practitioner, Prospective, Quality improvement

## Abstract

**Background:**

To better meet long-term care (LTC) residents’ (patients in LTC) needs, nurse practitioners (NPs) were proposed as part of a quality improvement initiative. No research has been conducted in LTC in Québec Canada, where NP roles are new. We collected provider interviews, field notes and resident outcomes to identify how NPs in LTC influence care quality and inform the wider implementation of these roles in Québec. This paper reports on resident outcomes and field notes.

**Methods:**

*Research Design:* This mixed methods quality improvement study included a prospective cohort study in six LTC facilities in Québec. *Participants:* Data were collected from September 2015–August 2016. The cohort consisted of all residents (*n* = 538) followed by the nurse practitioners. Nurse practitioner interventions (*n* = 3798) related to medications, polypharmacy, falls, restraint use, transfers to acute care and pressure ulcers were monitored. *Analysis:* Bivariate analyses and survival analysis of occurrence of events over time were conducted. Content analysis was used for the qualitative data.

**Results:**

Nurse practitioners (*n* = 6) worked half-time in LTC with an average caseload ranging from 42 to 80 residents. Sites developed either a shared care or a consultative model. The average age of residents was 82, and two thirds were women. The most common diagnosis on admission was dementia (62%, *n* = 331). The number of interventions/resident (range: 2.2–16.3) depended on the care model. The average number of medications/resident decreased by 12% overall or 10% for each 30-day period over 12 months. The incidence of polypharmacy, falls, restraint use, and transfers to acute care decreased, and very few pressure ulcers were identified.

**Conclusions:**

The implementation of NPs in LTC in Québec can improve care quality for residents. Results show that the average number of medications per day per resident, the incidence of polypharmacy, falls, restraint use, and transfers to acute care all decreased during the study, suggesting that a wider implementation of NP roles in LTC is a useful strategy to improve resident care. Although additional studies are needed, the implementation of a consultative model should be favoured as our project provides preliminary evidence of the contributions of these new roles in LTC in Québec.

## Background

Sixteen percent (1.5 billion) of the world’s population will be 65 years or older by 2050 [1]. Because countries must respond to increasing needs of this population, innovative approaches are needed to improve long-term care (LTC) services [2]. Furthermore, as healthcare systems internationally continue to face workforce issues, including shortages of physicians and other care providers in LTC, decision-makers are looking to shift care activities from physicians to nurses and nurse practitioners (NPs) to improve access to and the quality of LTC [3–6].

Care delivered by interprofessional teams where all roles are optimized represents an effective way to provide services [7]. In Canada, a national report on innovations in healthcare emphasized that expanding the role of NPs represented a potential solution to the healthcare workforce issues in primary care settings including LTC [8]. Donald et al. [9] surveyed NPs in LTC across Canada, and found that they assessed residents, managed chronic and acute illnesses, assisted nursing staff to develop new knowledge and skills, and coordinated care with residents, families and staff. In addition, NPs in LTC increase the level of knowledge of healthcare team members [10].

Globally, the LTC workforce has doubled since 2000 and represents 2 % of the healthcare workforce [11]. Internationally, authors have identified issues with access and funding in LTC [12–15]. In Canada, each provincial/territorial government manages its own health and social care, and inequities in care have been identified across the country [16]. Provinces and territories across Canada are struggling to meet current care needs for seniors [17]. For example, in Québec in 2011, 49 to 84% of seniors required an emergency room visit while awaiting LTC placement [18]. This trend is expected to worsen as the number of seniors requiring LTC in Québec is projected to double between 2011 and 2031 [19].

The Québec government will add 2000 NPs by 2024 to improve access to care in various settings, including LTC. NPs have graduate-level university education and experience. They monitor acute and stable chronic conditions, prescribe and monitor medications and tests [20, 21]. Evidence from international studies, including systematic reviews, has shown that NPs increase access to and the quality of services in LTC [4, 6, 10, 22], and they support nursing personnel to develop their knowledge and skills [23]. NPs improve patients’ and families’ satisfaction with care, and increase the use of advanced directives in LTC [22, 24–27]. Moreover, timely interventions and follow-up by NPs decrease unnecessary transfers of LTC residents to Emergency rooms by two thirds [28]. No research has been conducted in LTC in Québec, where NP roles are new, we have a limited understanding of how to support optimal implementation of NP roles in LTC.

Internationally, LTC services are provided in homes that are publicly- or privately- owned [29]. In Canada, publicly-funded facilities are accredited by a national body, whereas privately owned facilities do not have such requirements [30]. In Québec, where most (83%) LTC services are publicly funded [19], the Ministry of Health and Social Services determines staffing, services, and the criteria to admit residents. In privately owned and operated homes, the owners determine criteria to admit residents as well as staffing and service provision [31].

Within the European Union, the number of LTC beds relative to population size increased between 2010 and 2015 in most countries, with 1200 to 1300 beds per 100,000 inhabitants [32]. In Québec, however, the number of LTC beds has decreased in recent years to 459 beds per 100,000 inhabitants, and the rate of admission to LTC for those aged 65 years and older is 2.2%, which is lower than many other countries [32, 33]. In 2011, Québec instituted provincial policies to fund home care services to support seniors to remain in their home as long as possible [34], meaning that elderly patients are admitted to LTC with greater physical and cognitive impairments because they remain at home longer [19, 35]. Between 2011 and 2016, the length of stay of residents in LTC in Québec decreased by 11% (107 days). The most recent statistics indicate that the number of patients awaiting transfer (*n* = 2500) to LTC and the wait time for admission have remained stable (approximately 10 months) in the province [19, 36].

Studies indicate that how care is delivered impacts outcomes [37–39]. Several factors have been shown to influence NP practice and resident outcomes include physician remuneration, oversight, employment status, and NP decision-making autonomy [6, 40, 41]. In Québec, almost all NP positions are unionized, and salaries are paid by the Ministry of Health and Social Services, and most physicians are paid on a fee-for-service basis.

Internationally, models of care include NP-physician collaborative models [42, 43], NP co-management [44], physician substitution [3], and NPs as Most Responsible Providers [45]. Some authors have distinguished between a substitute role where NPs provide the same care as physicians and supplementation where NPs provide additional services to residents [3, 46]. Dahrouge and colleagues distinguished between the consultative and shared care models [47]. In the consultative model, NPs autonomously ensure the care of residents for activities and decisions within the scope of their field of expertise. They consult physician partners as needed, when the resident’s condition changes or care activities exceed their field of expertise. In the shared care model, NPs provide direct care and monitor residents’ emerging care needs without being assigned to care for a specific group of residents. Researchers have shown that the consultative model should be favoured because it leads to better outcomes for residents [48]. We retained Dahrouge et al.’s distinction because it allowed us to capture how NP contribute to care.

Indeed, the implementation of NP roles is complex [49], with factors including role clarity, support from managers and healthcare team members, clear messages from medical and nursing leaders on role priorities influence role implementation, team functioning, and care outcomes [50–52]. Since no research has been conducted in LTC in Québec, we aimed to support optimal implementation of NP roles in these settings.

### Conceptual framework

The conceptual framework of NP role enactment, boundary work, and perceptions of team effectiveness supported the project [53]. How roles are put in place, the process of changing boundary lines between professionals, and how teams function are at the centre of the framework. Structures from the patient to the healthcare system influence these processes. Moreover, structures and processes influence results, namely care quality, safety, costs, and team functioning. We operationalized how NP roles are implemented by examining the model of care and NP decision-making autonomy. Structures include resident, care provider, and LTC setting characteristics. Resident outcomes include medication, polypharmacy, falls, restraint use, transfers to acute care, pressure ulcers, and deaths.

## Methods

### Aims

The quality improvement study aimed to identify how NP roles influence care quality for residents in LTC to inform the wider implementation of these new roles in Québec. This paper reports on the findings from field notes and a prospective cohort study of residents receiving NP care. Findings from the qualitative descriptive study describing the views of providers and managers are reported elsewhere [54].

### Research design

A mixed methods quality improvement study was conducted [55, 56]. The Standards for Quality Improvement Reporting Excellence (SQUIRE 2.0) reporting guidelines were used with attention paid to describing the local context of implementation [57, 58]. We adopted the definition of a cohort proposed by Dekkers, Egger, Altman, and Vandenbroucke [59], and Mathes and Pieper [60]. A cohort is constituted of patients who are included based on an exposure (i.e., care provided by NPs), and who are followed-up to assess outcomes over time [59, 60]. These authors further argue that a comparison group is not a distinguishing feature of this type of study. The cohort was constituted using all residents (*n* = 538), followed by the NPs across six sites. NP interventions (*n* = 3798) indicative of high care quality in LTC (i.e., medications, polypharmacy, falls, restraint use, transfers to acute care, pressure ulcers) were monitored prospectively over a 12-month period.

### Context of implementation

The research team collaborated with several stakeholders beginning in 2013 (i.e., university-affiliated teaching hospital, Ministry of Health and Social Services, nursing and medical regulators, and university) to introduce NPs in LTC and improve access to care. Because NP roles in LTC were not yet recognized in Québec at the time, the nursing regulator developed guidelines to support the implementation [61].

Sites volunteered to participate in the project and were selected because they anticipated a physician shortage in the next 12 to 18 months due to physician retirement or maternity leave. The NPs worked in primary care and were new to the LTC sites. Only one NP worked in primary care with the same physician partners in LTC (Site 1). From January to May 2015, monthly meetings were held with decision-makers, nursing and medical regulators, managers, and NPs to: 1) identify the need for NP implementation in their region; 2) define the NP roles to implement; 3) identify the existing resources and those to develop (e.g. offices, communications with teams) to support NP role implementation in LTC; and 4) identify a site coordinator in each facility who would collect indicator data.

For the period from May to September 2015, a process for choosing common indicators and obtaining resident functional autonomy scores (i.e., Iso-SMAF described below) from residents’ health records was identified in cooperation with the sites and the Ministry of Health and Social Services. The NPs integrated into the LTC homes over the summer of 2015 on a part-time basis (half-time) to facilitate recruitment into the project. Issues related to NP implementation and data collection for the indicators were discussed with participants at monthly teleconferences over a 24-month period.

The NPs were initially trained as primary healthcare NPs. To support the development of knowledge and skills required in LTC, the Ministry of Health and Social Services offered 35 h of theoretical and practical training prepared by experts in the field, with discussions of case studies involving LTC residents, the mission of LTC, pain, end-of-life care, optimal use of medications, clinical examination, assessment of mental status including dementia and cognitive disorders, and the behavioural and psychological symptoms of dementia. The training took place in September–October 2015.

### Settings

The six sites were located in four administrative regions of Québec. Site 1 was a 96-bed rural facility where the NP had a caseload of 76 residents. A laboratory for blood tests, X-rays, and an emergency room were available on site. Site 2 was a 145-bed urban facility where the NP had a caseload of 60 residents. This site also had blood tests and X-rays available on-site. Site 3 was a 128-bed urban facility where the NP had a caseload of 64 residents in two clinical units. Site 4 was a 174-bed urban facility where the NP had no established caseload, instead caring for residents as their needs arose. Site 5 was a 132-bed rural facility where the NP had a caseload of 25 residents, as well as respite and palliative care responsibilities. Site 6 was a 101-bed urban facility where the NP had a caseload of 25 to 35 residents in three care units, in addition to responding to residents’ emerging needs.

### Indicator data for the reference year

A survey was conducted in May 2015 with participating sites to ensure the availability of indicator data that was common to all sites for the reference year (June 1st, 2014 to May 31st, 2015), and that we could compare for outcomes before and after NP role implementation. Participants expected to have access to data regarding numbers of and reasons for transfers to acute care, average number of medications, average number and duration of restraints, pressure ulcers, falls, and the level of satisfaction of residents and their family members. Yet, although the research team attempted to obtain this information on several occasions, these data were no longer available at the time of data collection due to a major restructuring of the healthcare system in March 2015 and the retirement of several experienced managers and site coordinators. Since we had planned to examine differences in frequencies and percentages pre- and post- implementation, new analysis strategies were identified and are described below.

### Data collection

Data were collected from September 1st 2015 to August 31st 2016. The indicator extraction checklist and a user’s guide with rules for collecting data were common to all sites. All tools were pilot-tested with the sites in July and August 2015, then minorly adjusted using their feedback. Since the research team did not have access to resident health records, site coordinators created alpha-numeric codes for the data they abstracted weekly for residents who received care from NPs. These anonymized spreadsheets were sent weekly to the research team (MJ) who collated the data and created a database for each site. Site coordinators indicated start and end dates for all entries to specify the duration of each NP intervention. The abstractors identified who (physician or NP) initiated or changed a prescription or an intervention. These data were used to determine the model of care. As proposed by Dahrouge et al. [48] we attributed the consultative model if 70% or more of the interventions were initiated by the NP for that site. In the other cases, we attributed the shared care model. Field notes, documenting all teleconferences and study-related decisions were circulated to study participants and reviewed for accuracy.

### Instruments

To meet participants’ information needs, we collaboratively developed a user guide with an interactive data entry spreadsheet. For example, whenever possible, we used a drop-down menu for chart abstractors to select response options. The spreadsheet contained 1) organizational variables (e.g. region, site, site coordinator, date of data entry, identification number of each entry; who initiated the intervention [NP or physician]); 2) patient variables (e.g. age, sex, primary diagnosis, functional autonomy score for the past year [Iso-SMAF]); and 3) quality of care indicators.

Iso-SMAF assessments, standardized resident evaluations used across Québec since 2000, examine the level of functional autonomy of individuals to determine the social and healthcare services that are required [62]. Their use is mandated by the Ministry of Health and Social Services, and they are part of usual care provided to all residents in Québec. The Iso-SMAF assessments are completed annually by members to the interprofessional team following extensive 4-day training sessions [63]. Scores range from 1 to 14, with scores 11 or above indicating very poor motor and cognitive functioning [64]. Residents in LTC settings score above 10 [62].

At the outset, participants agreed that the aim of the project was not to document all NP activities but rather to document only NP interventions that represented a change in residents’ care. Although each intervention related to an indicator was documented as a separate entry on the data abstraction form, additional information could be added in a comment box.

### Operationalization of the variables

Medications were monitored using the number of medications per day per resident. Interventions related to medications were documented using the date of the change in the number of medications. Changes in medication were documented using the date of the prescription (not the date the prescription was filled). Chart abstractors used the records provided by the organization’s pharmacists to indicate the type and dose of the medication. Medication taken regularly and occasionally were included. Medications that were prescribed as needed but not taken were not counted. All administration routes were included. Nutritional supplements were excluded. Falls were documented using the date of the fall. Falls and restraint use require mandatory reporting to the Ministry. We included the number of residents who fell, the number of falls per day if the resident fell more than once. We documented falls when the NPs needed to intervene or if the resident was injured in any way. Restraints included the number of residents with restraints and type (e.g., lap belt, geriatric table) and duration (date at the beginning and end of application) of each restraint. The date and time of application and removal were included. With transfers, the site coordinators documented the date and hour of the departure and return of residents, the reason for and duration of transfers to acute care, and date and time of the definitive transfer to another institution. Pressure ulcers ≥ stage 2 were documented and included the number of residents with a pressure ulcer, ulcer stage, the number of ulcers and the duration. The number of respite care visits, number of residents requiring respite care, and deaths (in LTC facility, following transfer to acute care) were documented.

To conduct the analysis of occurrence, period and incidence were defined as follows:

- **Period:** Each period lasted 30 days. The 12 consecutive periods were exclusive and complementary. Given that the NPs integrated each LTC site over the course of the summer and already cared for some residents, Period 1 documented the incidence at the start of the study.
- **Incidence:** Ratio of the number of interventions for each indicator (i.e., indicator-interventions) in a given 30-day period to the number of residents for the same period.

$$ \mathrm{Incidence}=\frac{\mathrm{Number}\ \mathrm{of}\ \mathrm{new}\ \mathrm{indicator}-\mathrm{interventions}\ \mathrm{in}\ \mathrm{a}\ \mathrm{given}\ 30\mathrm{day}\ \mathrm{period}}{\mathrm{Number}\ \mathrm{of}\ \mathrm{residents}\ \mathrm{for}\ \mathrm{the}\ \mathrm{same}\ \mathrm{period}} $$

Two types of incidences were calculated and included incidence of indicator-interventions and incidence of change. The incidence of indicator-interventions, based on the resident’s date of admission and the start date of the intervention, was used to determine the incidence of transfers, falls, restraints and ulcers. The incidence of change reflects the time to change for an indicator-intervention based the resident’s date of admission, the start date of the indicator-intervention, and the date that the previous intervention ended. It was used to examine the time to change in the incidence of transfers and changes in the number of medications.

The number of residents was calculated based on the number of admissions and departures (death, respite, definitive transfers) for each period where:

n_0_ = number of residents present on September 1st 2015; and

p = period


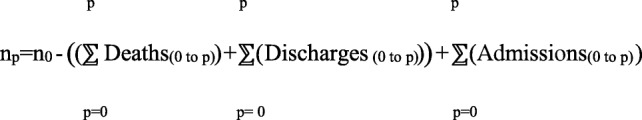


In a few instances (8%), the date of admission was unavailable for residents admitted before the start of the study, and whose first volumes of their health records were archived. In these cases, the date of their first prescription for medication was used as the best proxy for their date of admission. For residents admitted before September 1st 2015, these dates were corrected to begin on September 1st 2015. For residents who had indicator-interventions begin prior to August 31st 2016 and end after August 31st 2016, we imputed an end date of August 31st 2016.

#### Polypharmacy

There is no clear consensus in the literature on how to define polypharmacy [65, 66]. We adopted the definition proposed by Maher, Hanlon and Hajjar [67], which suggests that polypharmacy occurs if the average number of medications is nine or above. We therefore identified the number of residents who took nine or more medications at least once during each period. As proposed by Sirois and Émond [66], we opted for a cumulative count of a resident’s medication and identified medications taken regularly for chronic conditions and those taken sporadically to account for changes in residents’ health status. A cumulative count including both chronic and sporadic use of medications generates a higher prevalence of polypharmacy but also reveals the actual use of medications and exposure to polypharmacy [66].

### Analysis

Data sources (i.e., quantitative and qualitative) were analyzed separately for each site and subsequently combined across the six sites at the end of the study to understand how NPs influenced residents’ care quality [68]. The quantitative data was analyzed using IBM SPSS Statistics version 23 [69]. In generating descriptive statistics, frequencies and percentages were used for the categorical variables, while averages, standard deviation, and minimum-maximum were used for the continuous variables. Bivariate analyses generated trend curves (non-adjusted) of average medications per day per resident, polypharmacy, the incidence of transfers, falls, restraints, and pressure ulcers [70]. The qualitative data from the field notes were analyzed using content analysis [71].

#### Ethical considerations

Research ethics approval was not required for the quantitative phase of the quality improvement project in accordance with the 2014 Tri-Council policy statement [72]. The quantitative data were anonymized to protect residents’ identities.

#### Rigour

Several measures were taken to optimize data quality. To facilitate data collection and follow-up, and reduce the risk of recall bias, data files documented care 1 week at a time. Each file was sent to the research team and reviewed line by line by one research team member (MJ) and the site coordinator to ensure accuracy. Data abstractors were trained prior to study initiation, and the research team was available throughout the study to answer questions. Individual telephone and email follow-ups were scheduled at least once a week between the research team (MJ) and site coordinators to review the data files and answer questions. This continuous process allowed the research team to review inconsistencies, standardize data collection across sites, and identify missing data. Moreover, monthly teleconferences with all project participants provided an opportunity to answer questions.

## Results

From the qualitative data, the monthly meetings allowed us to ascertain that all NPs provided in-depth physical and mental assessments of residents, monitored and adjusted medications and treatments, and provided ongoing chronic illness care depending on the resident’s condition. All NPs collaborated with physicians to optimize resident care and determine care priorities. They supported the healthcare teams to use their assessment skills and clinical judgement to develop and update inter-professional care plans. Healthcare providers and managers noted that the NPs spoke regularly with family members to keep them informed of care priorities, changes in treatment plans, or in the resident’s condition.

**Fig. 1:**
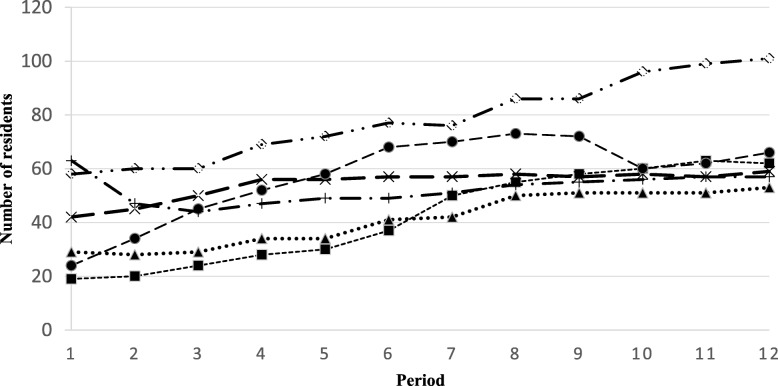
Number of residents cared for by the nurse practitioners per period

Using the quantitative data, we found that the NPs cared for 538 residents across the six sites in the first 12 months of implementation. The average age of residents was 82 (SD: 11 years, range: 24 to 103 years), and the average functional autonomy score was 11 (range 3–14). Almost two thirds of the residents cared for were women (62%, *n* = 333). The most common diagnosis on admission was dementia (62%, *n* = 331) followed by cardio-vascular disease (12%, *n* = 62) and neurological disorders (10%, *n* = 53). The number of residents cared for by each NP ranged from 42 to 80 (Fig. 1). The average number of interventions per resident ranged from 2.2 to 16.3, depending on the care model (Table 1).

**Table 1:** Model of care and number of nurse practitioner and physician interventions per resident at the end of the study

Site	Model of Care	Residents	Number of Interventions		Interventions/ Resident					Number of Medications				
		n	NP^a^	MD^b^	Mean	SD^c^	Range	Confidence Interval		Mean	SD	Range	Confidence Interval	
1	Consultative	104	853	0	8.2	5.8	1–39	7.1	9.3	9.8	3.8	4–19	9.1	10.5
2	Shared Care	61	81	508	9.7	7.6	1–34	7.8	11.6	12.5	5.1	4–29	11.2	13.8
3	Shared Care	73	221	18	3.3	1.9	1–9	2.9	3.7	13.9	5.9	4–30	12.5	15.3
4	Shared Care	108	11	231	2.2	1.6	1–7	1.9	2.5	12.2	5.3	3–28	11.2	13.2
5	Consultative	90	138	70	2.3	1.6	1–9	2.0	2.6	11.8	4.7	2–31	10.8	12.8
6	Shared Care/ Consultative	102	847	820	16.3	12.5	1–49	13.9	18.7	11.5	4.5	4–6	10.6	12.4
Total		538	3798		7.1	8.4	1–49	6.4	7.8	11.8	5	2–31	11.4	12.2

^a^*NP* Nurse practitioner, ^b^*MD* Physician, ^c^*SD* Standard deviation

Over the course of the study, the consultative model was used at sites 1 and 5; the shared care model was used at sites 2, 3 and 4. Field notes indicate that a mixed care model was implemented at site 6. The NP moved to a consultative model after the physician partner returned from maternity leave.

The number of residents cared for by NPs generally increased during each 30-day period, reaching more than 60 residents at sites where there was sustained cooperation from physician partners (Fig. 1). However, there was a decrease in the number of residents cared for by NPs that coincided with the departure of one physician partner during period 2 at site 6 and period 10 at site 5. The number of residents cared for by NPs at sites 5 and 6 was lower than at the other sites because physician partners either retired or were on maternity leave, and no additional physician agreed to replace them. The NPs developed an additional admission service, respite for family members, at sites 5 and 6, resulting in additional evaluations and care activities. Moreover, the NP at site 5 resumed care for residents once the physician partner returned from maternity leave.

### Indicator monitoring

**Fig. 2:**
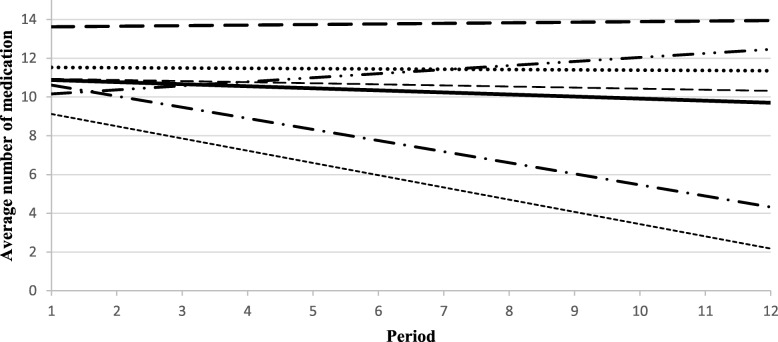
Trends in the average number of medications per resident per period

**Fig. 3:**
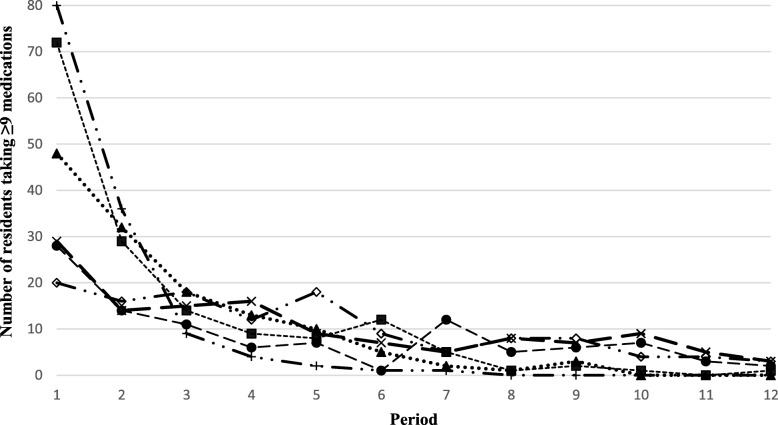
Polypharmacy among residents followed by the nurse practitioners per period

At the end of the study, the average number of medications per day per resident ranged from 9.8 (SD: 3.8) to 13.9 (SD: 5.9) depending on the site, and decreased by 10% for each 30-day period over 12 months (or 12% overall) (Table 1). The average number of interventions related to medication ranged from 1.8 (SD: 1.1) to 8.7 (SD: 7.1). A decrease in the average number of medications was observed at four of the six sites (Fig. 2), with the largest decreases noted at sites where NPs 1) regularly monitored residents’ medication; 2) made autonomous decisions regarding activities in their field of expertise; and 3) collaborated more extensively with pharmacists. An increase in the average number of medications was noted at site 4 because the NP dealt primarily with residents’ emerging needs (e.g., agitation, infection requiring prescription medication) and did a limited number of follow-ups with regards to residents’ needs. There was also a considerable decrease in polypharmacy rates at several sites in the first 2 months after the NP ensured the follow-up of residents (Fig. 3).

**Fig. 4:**
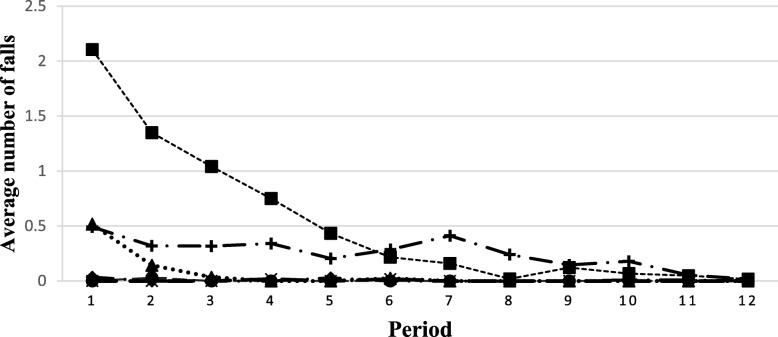
Trends in the average number of falls per resident per period

**Fig. 5:**
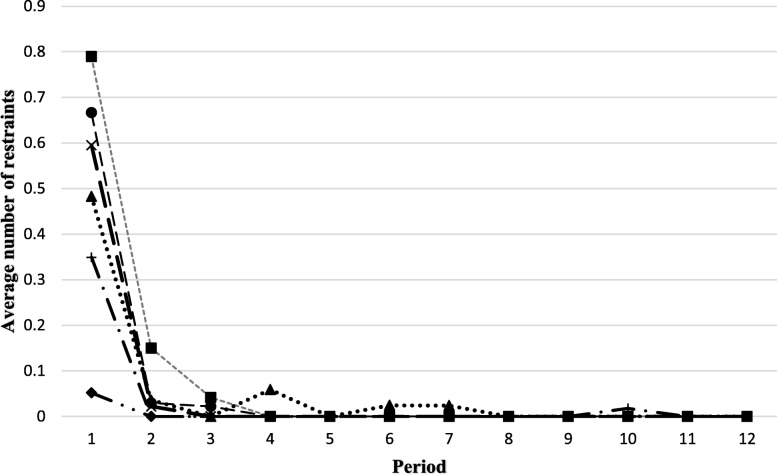
Trends in the average number of restraints per resident per period

**Fig. 6:**
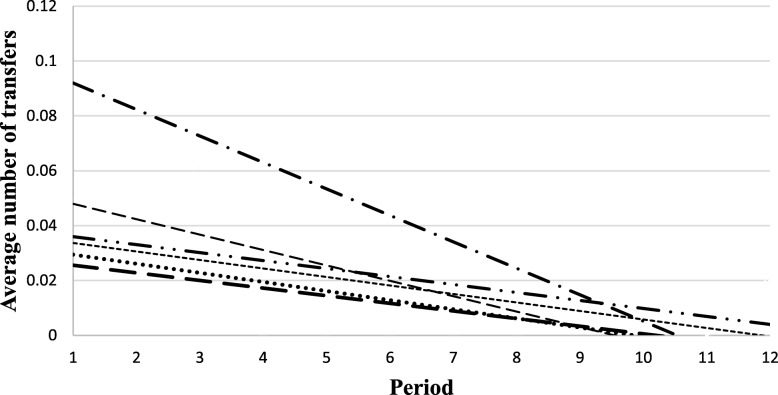
Trends in the average number of transfers per resident per period

**Fig. 7:**
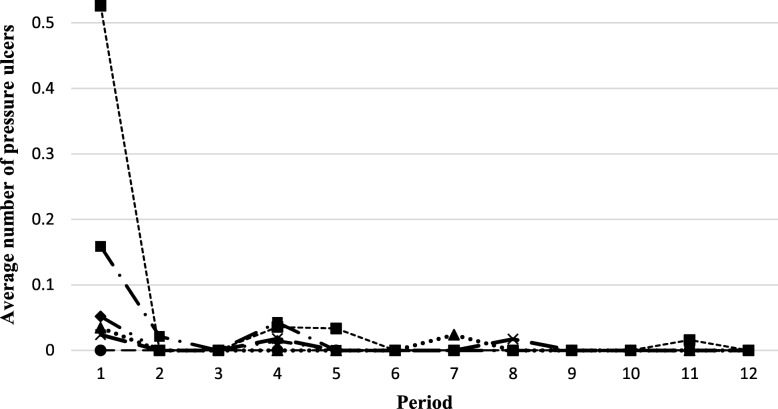
Trends in the average number of pressure ulcers per resident per period

Considerable variation in the incidence of the other indicators was noted at each site (Figs. 4, 5, 6 and 7). The use of restraints varied by site and reflected the practices and policies in place at each site. However, a pattern was identified where a decrease in the use of restraints was noted after the first period, and this number subsequently remained low for all sites (Fig. 5). Very few new pressure ulcers were recorded (Fig. 7). The number of transfers to acute care ranged from 4 to 24, and the duration of the transfers ranged from a few hours to almost 9 days, depending on the site. Two LTC facilities were located in buildings that housed regional hospitals where they continued to have access to X-ray technology and laboratories for blood tests. This decreased the transfer rate to acute care for these sites. Reasons for resident transfers included X-Ray examinations, consultations with specialists subsequent to a fall, or a sudden change in condition that led to death while in acute care. Across all sites, the incidence of transfers to acute care decreased over time.

## Discussion

This quality improvement project aimed to support the implementation of NP roles in LTC in Québec Canada and determine how they influenced care quality. The NPs worked part-time in LTC. They provided in-depth of assessments of residents’ conditions, prescribed and monitored medications and treatments, worked with members of the healthcare team to develop their knowledge and skills, and involved residents and families in care decisions. NP caseload increased over the course of the study and ranged from 42 to 80. The number of medications decreased by 12% by the end of the study. Factors including NP decision-making autonomy for activities in their field of expertise, and collaborations between NPs, physician partners, pharmacists and other members of the healthcare team had a major impact on the care model that was developed and resident outcomes. All these factors were present at Site 1 over the course of the study where quality indicators improved markedly giving a preliminary indication that the more modest improvements we saw at the other sites could be significantly enhanced with a more sustained implementation of the consultative model for NP roles. One site (site 4) was different from the others as the physician partner wanted to maintain a shared care model at the outset of the project. The results for this site shed some light on the influence of the shared care model on resident outcomes. Overall, our project highlights the complex nature of NP role implementation, adding new knowledge about NP contributions to care quality, NP caseload and how caseload evolves over the first year of implementation in LTC.

For our study, having NPs work part-time in LTC greatly facilitated our NP recruitment. When Klaasen et al. [73] undertook a quality improvement study to reduce the number of transfers to the Emergency department where the NP worked full-time using a consultative model, they also documented improved outcomes for residents. However, their recruitment of a full-time NP required two years.

Internationally, our study aligns with others that have shown that NPs improve care quality for LTC residents and promote the development of knowledge among healthcare providers [5, 22, 23, 41, 74, 75]. NPs in our study cared for residents with complex care needs with characteristics that were similar to other LTC residents in Québec with regards to age, sex, diagnosis on admission, and functional autonomy scores [19, 31, 76]. Our findings are also consistent with a recently completed scoping review by Chavez et al. [6] who examined interventions performed by NPs in LTC and their impact on patient and financial outcomes, finding that NPs improved care quality despite sparse evidence examining costs. Santosaputri et al. [74] completed a systematic review examining factors that influence hospital avoidance for residents in LTC, and found that healthcare professional decisions to transfer residents to Emergency departments are complex and include multiple factors. Our study adds new knowledge by identifying the NPs’ contributions in reducing the number of transfers to the Emergency department. Subsequent analyses will allow us to estimate costs related to reductions in medications, falls, restraint use, transfers to acute care, and pressure ulcers. Furthermore, the prospective nature of data collection limited the risk of recall bias in our project as we lost no patients to follow-up and all subjects had complete outcome data, limiting attrition bias [77, 78].

Polypharmacy is a major problem among the elderly. In Canada, approximately 40% of seniors take potentially dangerous medications [79]. Professionals, patients, and families have different priorities when it comes to deprescribing potentially dangerous medications in LTC [80]. A study in Australia showed that physicians and pharmacists were in agreement in only 44% of cases when it came to discontinuing medications specific to seniors in LTC [81]. Liu [82] believes that such differences can be attributed to healthcare professionals’ philosophy of care, time needed to do an in-depth analysis of residents’ medications, and lack of explicit criteria for discontinuing certain medications among seniors in LTC. NPs can play a key role in managing LTC residents’ medications through their extensive knowledge of resident and family priorities, their holistic vision of care, and increased presence in LTC.

Although the conceptual framework [53] we used was initially developed to understand processes in healthcare teams with NPs in acute care, it allowed us to operationalize structural, process, and outcomes variables and relationships included in the framework and apply them in LTC [83]. Additional research using controlled studies or experimental designs is needed to further refine our understanding of these relationships.

O’Rourke and Fraser [56] completed a systematic review to examine how quality improvement projects advance knowledge for research and practice, highlighting that rigorous evaluations are needed to strengthen the knowledge base. In particular, they argued that quality improvement projects often focus on a single site, using pre- and post- implementation measures, and instead suggested that in-depth descriptions of the context and how the intervention was implemented reduce the risk of bias. Thus, our study adds new knowledge by examining practices in several sites and providing in-depth descriptions of the context of implementation.

Subsequent work must address the sustainability of these new roles in LTC in Québec. Project findings have already been used by regulators and decision-makers in Québec to inform new laws that were passed in 2018. These laws expand the scope of practice of NPs, allowing them to practice in LTC. However, the lack of data available for the year prior to implementation is of concern. According to the Canadian Association for LTC [84], several countries have made progress on the use of Minimum Data Set (MDS), Resident Assessment Instrument Minimum Data Set 2.0, and Management Information Systems. Several jurisdictions in Canada are lagging behind their European counterparts and no facility in Québec collects these data. Clearly, given the increase in the need for LTC services, better information systems are required to support evidence-informed decisions and proper resource allocation in LTC.

Some limitations need to be kept in mind. This project was undertaken as a quality improvement study to account for the complexities of NP role implementation. Our inability to access data for the year prior to implementation limits the comparison of resident outcomes before and after implementation and highlights some of the challenges in conducting quality improvement projects where health records are paper-based. The revised analysis strategy allowed us to identify a pattern in residents’ care in the first two months of NP follow-up [56]. Additionally, the performance of teams who volunteered for the project is probably not typical of most teams in LTC. Nor was the point of view of residents and their families assessed in this project. A more in-depth examination of their views is needed to guide the implementation of NPs in LTC in Québec, as researchers in other jurisdictions have found that residents and families appreciated the addition of NPs to LTC teams [85].

We anticipate that our findings will be relevant to other jurisdictions given that we collected resident outcome data prospectively, and the information gleaned from the sites represented essential elements to understand how to implement NP roles in LTC. With the optimization of roles within healthcare teams and the improvement in the quality of follow-up, LTC teams that include NPs can become more attractive work environments for healthcare providers and desirable student trainee sites. Further studies are needed to examine the financial impact of these roles in LTC. To ensure a more precise estimate of the effect of implementing NPs and control for confounders, future studies should include the random assignment of residents to intervention and comparison groups.

## Conclusion

This quality improvement project aimed to inform NP role implementation in LTC in Québec Canada. Results show that the number of medications decreased by 12% at the end of the study, and the incidence of polypharmacy, falls, restraint use, and transfers to acute care also decreased. The consultative model should be favoured as our project indicates that it provides preliminary evidence of the contributions of these new roles in long-term care in Québec. Even if controlled before and after or experimental studies are now needed, this project enabled a better understanding of the factors that influence nurse practitioner role implementation and resident outcomes in LTC. An in-depth examination of the views of residents and families can further guide the rollout of NP roles in LTC in Québec.

## Data Availability

The datasets used and/or analysed during the current study are available from the corresponding author on reasonable request.

## Abbreviations

CIUSSS-EMTL: Centre intégré universitaire de santé et des services sociaux de l’Est-de-l’Île-de-Montréal
LTC: Long-term care
NP: Nurse practitioner
SD: Standard deviation
